# Lactylation and antitumor immunity

**DOI:** 10.3389/fimmu.2025.1690068

**Published:** 2025-10-16

**Authors:** Biao Yang, Lingyu Li, Dongmei Shi, Tao Zhong, Huabao Xiong

**Affiliations:** ^1^ The Laboratory of Medical Mycology, Jining No. 1 People’s Hospital Affiliated to Shandong First Medical University, Jining, Shandong, China; ^2^ Institute of Immunology and Molecular Medicine, Key Laboratory of Cell and Biomedical Technology of Shandong Province, College of Basic Medicine, Jining Medical University, Jining, China

**Keywords:** lactylation, antitumor immunity, lactate accumulation, tumor microenvironment, histone and non-histone lactylation, immunosuppressive phenotypes

## Abstract

Lactylation, a recently discovered post-translational modification (PTM), plays a critical role in cancer biology. Warburg effect induces lactate accumulation, which serves as a metabolic end-product and intercellular signaling mediator within the tumor microenvironment (TME). Beyond fueling tumor growth, elevated lactate levels drive histone and non-histone lactylation, which modulates gene expression and protein function. This epigenetic reprogramming induces immunosuppressive phenotypes in immune cells that are resident in the tumor microenvironment, including impaired effector function, enhanced immunosuppressive cytokine secretion, and altered tumor antigen presentation, collectively facilitating immune escape. This review provides a synthesis of the current understanding of lactate and lactylation in tumor immunosuppression, detailing molecular mechanisms underlying immune cell inhibition (tumor-associated macrophages, T cells, T-reg cells, NK cells and NKT cells, as well as neutrophils) and evaluating emerging therapeutic strategies (*e.g.*, inhibitors of MCTs/LDHA, site-specific antibodies, genetic code expansion technology). We aimed to accelerate the clinical translation of lactylation-targeted anticancer therapies by highlighting recent advances.

## 1 Introduction

Post-translational modifications (PTMs) alter proteins after translation, regulating structure, activity, localization, stability, and interactomes (1). These modifications critically regulate physiological processes and disease pathogenesis, making PTM research essential to understand biological mechanisms, identify clinical biomarkers, and discover therapeutic targets (2). Common PTMs include phosphorylation, acetylation, methylation, ubiquitination, succinylation, palmitoylation, and the recently identified lactylation (3). In 2019, Zhang et al. pioneered the discovery of histone lysine lactylation (Kla) in mammalian cells using tandem mass spectrometry and isotopic tracing (4). This finding established Kla as a bona fide PTM, unveiling new research dimensions in determining the roles of lactate in cancer, immunity, and metabolism.

Kla is the covalent conjugation of lactate to specific lysine residues via enzymatic or non-enzymatic mechanisms (5). Through this modification, protein function and transcriptional programs are modulated, crucially influencing cellular physiology, tumorigenesis, and immune regulation (6). The TME, comprising malignant cells, immune populations, vasculature, stroma, and signaling networks, critically determines tumor progression and therapeutic responses (7). Paradoxically, immune surveillance eliminates malignant cells; meanwhile, TME-imposed immunosuppression enables tumor immune escape (8). Consequently, deciphering immune cell functionality within the TME is fundamental for developing effective anticancer therapies.

Lactate, which was once considered a metabolic waste product, is now recognized as a key signaling molecule and metabolic regulator. In tumors, cancer cells preferentially use aerobic glycolysis, known as the “Warburg effect”, consuming excessive glucose to generate adenosine triphosphate (ATP) biosynthetic precursors while accumulating lactate (9). This metabolite subsequently shuttles energy and signals across TME compartments, coordinating metabolic symbiosis (10). Notably, lactate-derived Kla modifies histones and non-histones, thereby reprogramming gene expression and immune responses (11).

Accumulating evidence shows that the acidic milieu caused by the build-up of lactate within the TME directly impedes immune cell activation and proliferation, and concomitantly potentiates the functionality of immunosuppressive cells via Kla. This cascade precipitates tumor immune evasion through multifaceted mechanisms: functional impairment of effector immune cells, amplified secretion of immunosuppressive cytokines, upregulation of immune checkpoint molecules, and altered tumor-associated antigen presentation (12–14). Collectively, based on these, a profoundly immunosuppressive niche is established. Findings from empirical studies show that lactate concentration in the TME reaches 30–40 mM, exerting dual immunosuppressive mechanisms through microenvironmental acidification and direct molecular signaling (15). Elevated lactate concentrations suppress T-cell and natural killer (NK)-cell proliferation, cytotoxic activity, and interferon-γ (IFN-γ) secretion (16). Conversely, lactate promotes M2-polarization of tumor-associated macrophages (TAMs) (17) and myeloid-derived suppressor cells (MDSCs), while stimulating their secretion of immunosuppressive mediators, such as vascular endothelial growth factor (VEGF), interleukin (IL)-10, and accelerating programmed death−ligand 1 (PD-L1) expression (18). Lactate-driven Kla orchestrates extensive reprogramming of immune cells to augment TME immunosuppression. For instance, lactate-derived lactyl-CoA facilitates histone Kla (*e.g.*, at histone H3 lysine 18 lactylation [H3K18la]), thereby inducing immunosuppressive gene expression (including *Arg1*) and driving macrophage polarization toward the M2 phenotype (19). Furthermore, lactate enhances transforming growth factor (TGF)-β signaling in regulatory T cells (Tregs) through non-histone Kla modifications, exemplified by MOESIN Kla at lysine 72, which subsequently attenuates CD8^+^ T cell functionality (20, 21). Based on these observations, lactate and its associated Kla modifications are recognized as central regulators of tumor progression through immunosuppression. Emerging therapeutic strategies targeting lactate metabolism, including glycolytic inhibition, lactate transporter blockade, combination with immune checkpoint inhibitors, and epigenetic modulation, such as Kla enzyme inhibitors, could potentially aid in subverting the immunosuppressive TME, augmenting immune infiltration, and potentiating chemotherapeutic efficacy (22, 23). Nevertheless, challenges persist owing to the non-specificity of Kla-associated enzymes, cellular heterogeneity within TME subsets, and unresolved safety profiles of metabolic inhibitors. Consequently, elucidating the role of Kla in immune cells offers considerable therapeutic relevance for developing targeted antitumor immunotherapies.

In summary, these findings underscore the pivotal contribution of lactate and Kla to establishing immunosuppressive TME. In light of recent discoveries regarding lactate-mediated Kla in tumor immunology and immunotherapy, this review provides a synthesis of the current understanding of the immunomodulatory effects of Kla and the translational landscape of Kla-targeted therapeutics. We herein consolidate the research paradigm, historical advances, and future trajectories concerning lactate metabolism and Kla in TME-mediated immune suppression.

## 2 Lactate and lactylation

### 2.1 Lactate production

Glucose constitutes the ubiquitous primary nutrient source for cellular metabolism. Following cellular uptake, it is enzymatically converted to pyruvate via sequential catalytic reactions, yielding modest quantities of ATP and nicotinamide adenine dinucleotide (NADH) (24). Conventionally, glucose-derived energy production proceeds through two principal metabolic pathways—glycolysis and mitochondrial oxidative phosphorylation, both initiating from pyruvate. Under normoxic conditions, pyruvate and electron-carrying NADH translocate to the mitochondria, where pyruvate decarboxylation generates acetyl-CoA for entry into the tricarboxylic acid (TCA) cycle, culminating in robust ATP synthesis (25). Conversely, pathological hypoxia triggers the hyper-uptake of cellular glucose, leading to exclusive reliance on cytoplasmic glycolysis. Electron transfer constraints prevent the mitochondrial pyruvate from using pyruvate, diverting it to lactate via lactate dehydrogenase A (LDHA) catalysis. This anaerobic glycolytic pathway yields only 2 ATP molecules per glucose unit, precipitating substantial lactate accumulation (26). Notably, tumor cells exhibit aerobic glycolysis (the Warburg effect), a metabolic reprogramming wherein glycolysis dominates ATP production despite oxygen availability, rapidly generating ATP and lactate to fuel neoplastic proliferation (15).

**Figure 1:**
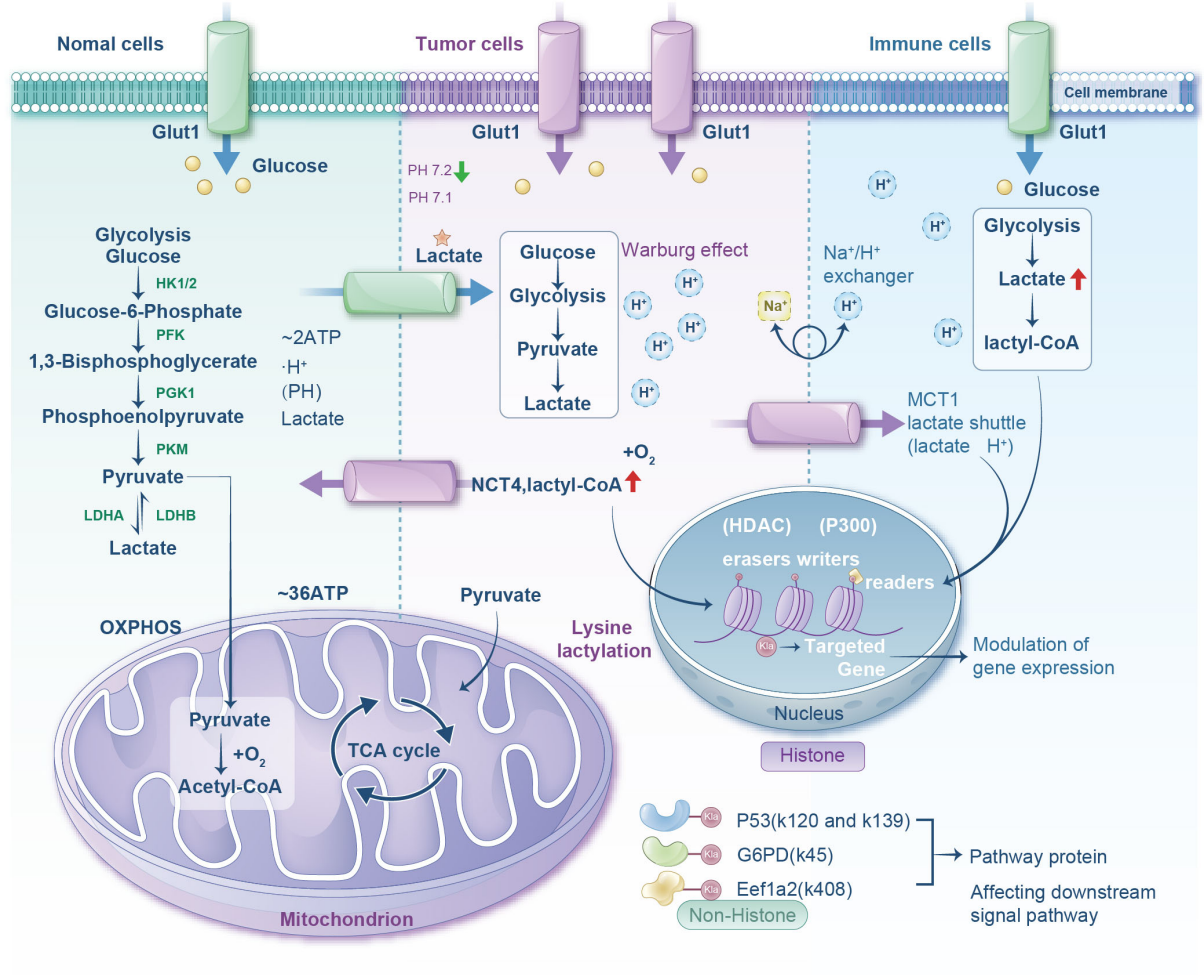
Diagram comparing metabolic processes in normal, tumor, and immune cells. It shows glucose transport via Glut1, glycolysis progression, and lactate production. Normal cells lead to mitochondrial oxidative phosphorylation. Tumor cells show the Warburg effect and lactate production with lactyl-CoA involvement. Immune cells exhibit similar glycolytic pathways with increased lactate and lactyl-CoA. The nucleus section highlights histone modifications affecting gene expression through lysine lactylation. Pathway proteins affected include P53, G6PD, and Eef1a2.

The Warburg effect modulates the TME through three interconnected mechanisms: signaling cascades, transcriptional regulation, and metabolic enzyme modulation. Hyperactivation of the PI3K-AKT-mTOR axis in malignancies promotes glycolysis via: (i) upregulation of glucose transporters, such as GLUT1, GLUT3 to enhance glucose influx; (ii) hexokinase (HK2) activation accelerating glucose phosphorylation and intracellular retention; and (iii) mitochondrial pyruvate carrier suppression, limiting pyruvate entry into mitochondria to attenuate oxidative respiration (27–29). Within the Ras-mitogen-activated protein kinase (MAPK) pathway, Ras activation induces extracellular signal-regulated kinase-mediated phosphorylation of transcription factors (*e.g.*, c-Myc), driving expression of glycolytic genes (including *HK2*, *PFK1*, *PFK2*, and *PKM2*) (30). Pyruvate kinase M2 may adopt a low-activity state through phosphorylation (*e.g.*, by AKT) or protein interactions (*e.g.*, with c-Myc), causing the accumulation of glycolytic intermediates (*e.g.*, fructose-1,6-bisphosphate) (31). These intermediates feed into ancillary pathways—notably the pentose phosphate pathway and one-carbon metabolism—to furnish nucleotide precursors (NADPH, ribose) supporting tumor proliferation (32). The hypoxia-inducible factor (HIF-1α) axis further orchestrates metabolic reprogramming. Persistent HIF-1α stabilization in malignancies arises from the accumulation of genetic lesions (*e.g.*, *VHL* deletion) or reactive oxygen species (ROS) under normoxia, compounded by tumor vascular abnormalities inducing regional hypoxia (33). In hypoxic tumor cores, activating HIF-1 prompts nuclear translocation and transcriptional upregulation of glycolytic machinery: glucose transporters (glucose transporter 1 (GLUT1)/GLUT3), HK2, PFK, and LDHA—collectively amplifying glycolytic flux (34). Synergistically, c-Myc potentiates HIF-1α transcriptional activity, establishing a feedforward regulatory loop. This cascade includes phosphorylation and inhibition of pyruvate dehydrogenase by PDK1, thereby obstructing pyruvate entry into the TCA cycle (35). Concurrently, LDHA and PDK upregulation coordinately divert pyruvate toward lactate production (36) (**Figure 1**).

Glycolytically derived lactate conversion is principally catalyzed by lactate dehydrogenase (LDH)—a tetrameric enzyme composed of LDHA or LDHB subunits. LDHA preferentially converts pyruvate to lactate, whereas LDHB favors the reverse reaction. Physiologically, lactate exists predominantly in its dissociated form (37). In humans, L-lactate is the predominant isoform (serum concentration: 1–2 mM), with D-lactate present at nanomolar levels. Lactate critically sculpts the TME: Aerobically generated lactate establishes localized acidic niches that perturb immune cell infiltration, fostering immunosuppression and tumor proliferation (38). Furthermore, monocarboxylate transporters (MCTs; *e.g.*, MCT1, MCT4) mediate lactate transmembrane shuttling, with MCT-facilitated lactate dynamics intimately associated with tumor pathophysiology.

### 2.2 Lactate shuttle

Lactate, the terminal glycolytic metabolite, functions as a versatile molecular shuttle—intracellularly, intercellularly, and systemically—modulating cellular bioenergetics and redox equilibrium while accumulating across tissues via circulatory transport (39). MCTs and LDHs orchestrate lactate exchange across plasma membranes, mitochondrial compartments, and the extracellular matrix. Under aerobic conditions, cells import extracellular lactate primarily through MCT1. This lactate pool may undergo mitochondrial translocation via MCT1 for TCA cycle oxidation or cytoplasmic reconversion to pyruvate by LDHB (40). Concurrently, glycolytic pyruvate is reduced to lactate by LDHA and extruded via MCT4, establishing an acidic extracellular niche (41). Hypoxic, hydrogen peroxide, and lactate stimuli further upregulate HIF-1 expression, which transactivates MCT4 to amplify lactate efflux (42). This coordinated shuttle system enables metabolic coupling between glycolysis and oxidative phosphorylation, optimizing tumor bioenergetic resource allocation.

The aforementioned glycolytic reprogramming drives profound lactate and proton accumulation. Paradoxically, while this should acidify the cytosol (intracellular pH, pHi), neoplastic cells maintain pHi within 7.1–7.7 (versus ~7.2 in normal cells) through adaptive mechanisms: (i) upregulated SLC16A3 (encoding MCT4) extrudes lactate/H^+^; (ii) CAIX (encoded by CA9) catalyzes CO2 hydration to bicarbonate/H^+^, buffering glycolytic proton burden; and (iii) hypoxia/acidosis-activated Na^+^/H^+^ exchanger 1 (NHE1) extrudes protons (29, 43, 44). Consequently, tumor extracellular pH (pHe) plummets to 6.7–7.1 (versus ~7.4 normally), creating an acidic microenvironment that potentiates metastasis, invasion, and immune evasion (45).

This lactate-forged acidic niche constitutes a “global protective shield,” subverting antitumor immunity. Cytotoxic T cells (CTLs) and NK cells—critical antitumor effectors—are functionally impaired and undergo apoptosis under acidic pHe conditions, crippling host defenses (45). Lactate shuttling within the TME exhibits dual pathological significance. First, lactate serves as glycolytic waste and oxidative substrate, fueling metabolic coupling between hypoxic and oxygenated tumor subregions (46). Second, elevated lactate directly inhibits immune cell activation/proliferation and drives Kla-mediated immunosuppression, promoting CTLA-4 expression in T cells, M2-like macrophage polarization, and dendritic cell dysfunction (47).

Therapeutically, Huang et al. engineered a pH-activatable nanomedicine targeting MCT1 to reverse lactate-induced immunosuppression. In their AZD-UPS nanoparticles, AZD3965 (MCT1 inhibitor) was encapsulated within ultra-pH-sensitive (UPS) polymers, maintaining micellar integrity at pH 7.4 but rapidly releasing payload in acidic TME. Combined with anti-programmed cell PD-1 immunotherapy, AZD-UPS NPs achieved superior tumor control and survival benefit at >200-fold lower dosage than oral AZD3965 monotherapy, demonstrating that MCT1 blockade can reshape the immunosuppressive TME to potentiate checkpoint inhibition (48) (**Figure 1**).

Collectively, lactate shuttling mechanics represent an oncological paradigm linking metabolic crosstalk to immune evasion. Deciphering this biology is extremely important to understand tumor immunosuppression and innovate next-generation therapeutics.

### 2.3 Lactylation modification

Since its inaugural characterization, Kla has become a pivotal PTM. Kla is formed through amide bond formation between the carboxyl group of lactate (CH_3_CHOHCOOH) and the ϵ-amino group of lysine, yielding N-ϵ-lactyllysine (Lys-lactate) (49). Analogous to acetylation and succinylation, Kla dynamics are governed by enzymatic “writers” (installers), “erasers” (removers), and “readers” (recognition modules) that collectively modulate protein function and stability (50). The lactyltransferase repertoire has been expanded in recent discoveries to include EP300/CBP, KAT7, KAT8, AARS1, and AARS2 (51). Conversely, documented erasers, including HDAC1–3, HDAC8, and SIRT1–3, exhibit broad substrate selectivity across multiple acyl-PTMs *(e.g.*, acetylation, methylation), lacking Kla specificity (52, 53).

Kla targets histones (*e.g.*, H3K18la) and non-histone proteins (*e.g.*, β-catenin, METTL16), regulating diverse biological processes through alterations in protein stability, activity, and interactome (54). Lactate, the common substrate for both pathways, exists as two isomers, L-lactate and D-lactate, with L-lactate participating in enzymatic Kla and D-lactate in non-enzymatic processes (38).

Using L-lactate as a substrate, solvent-exposed lysine residues exhibit nucleophilic reactivity owing to their ϵ-amino group’s chemical flexibility. Pathway bifurcation occurs via L-lactate conversion to lactyl-CoA, followed by p300/TIP60-mediated transfer to lysine or generation of lactate-AMP by AARS1/2, enabling direct lactyl group transfer (55). Driven by D-lactate accumulation during pathological states (*e.g.*, diabetes, dysbiosis), chemical reactivity enables spontaneous modification, potentiated by glyoxalase system dysregulation: methylglyoxal is converted by glyoxalase 1 (GLO1) to lactoylglutathione (LGSH), which undergoes non-enzymatic acyl transfer to lysine residues (55).

Cellular lactate concentration directly modulates Kla levels (glycolytic inhibition suppresses Kla, while mitochondrial dysfunction or hypoxia enhances it) (56). Following installation, specialized “reader” domains decode Kla signals to orchestrate downstream events. Ultimately, HDAC/Sirtuin erasers hydrolyze the lactyl-lysine bond to terminate signaling (57). Furthermore, the glyoxalase pathway regulates non-enzymatic Kla: GLO2 hydrolyzes LGSH to regenerate glutathione and produce D-lactate. Imbalanced GLO1/GLO2 expression diverts the flux toward LGSH accumulation, thereby accelerating non-enzymatic Kla (58) (**Figure 1**).

## 3 Lactylation and tumor immunosuppression

Immune cells within the TME engage in malignant cell surveillance through recognition and elimination pathways. Tumorigenesis triggers innate and adaptive immune activation; however, these malignancies develop multifaceted evasion strategies, including genetic, epigenetic, and metabolic reprogramming, to circumvent immune surveillance (59). Concurrently, tumors recruit and polarize immunosuppressive cell populations, actively sculpting an immune-permissive niche. Critically, glycolytically derived lactate extruded into the extracellular space functions as a metabolic intermediary and an immunomodulatory signal. Through Kla-mediated epigenetic rewiring, lactate actively suppresses antitumor immunity while potentiating oncogenesis (60, 61).

Kla operates as a lactate-dependent PTM that alters chromatin architecture and protein interactomes. Kla reprograms transcriptional networks regulating immune evasion by modifying histone tails (*e.g.*, H3K18la) and non-histone targets (62). This modification orchestrates TME immunosuppression through dual mechanisms by suppressing effector immunity by impairing cytosolic DNA sensing via cGAS activity attenuation and amplifying TGF-β signaling cascades in cytotoxic lymphocytes, while simultaneously enabling immune checkpoints by upregulating PD-L1 expression on antigen-presenting cells and stabilizing immunosuppressive Treg and TAM phenotypes (63).

Strikingly, elevated histone Kla correlates with advanced tumor grade/stage and TME remodeling—manifested as increased immunosuppressive infiltrates (Tregs, M2 macrophages) and checkpoint molecule overexpression (PD-1, CTLA-4, LAG-3) (64, 65). These clinical observations underscore the central role of Kla in coordinating tumor metabolic adaptation with immune escape pathways.

### 3.1 Macrophages

Macrophages have exceptional phenotypic plasticity, dynamically transitioning between pro-inflammatory (M1) and reparative (M2) states in response to microenvironmental cues. Modifying Kla serves as a metabolic-epigenetic switch that orchestrates this polarization by modifying key signaling nodes, including cytokine networks and immune response regulators (66). Notably, lactate accumulation reprograms macrophage bioenergetic pathways and drives their functional repolarization from pro-inflammatory to reparative phenotypes through shifts in intracellular metabolite availability (67).

This Kla-mediated immunometabolic reprogramming fundamentally alters macrophage behavior in the TME. Through the modulation of the effector functions of TAMs, Kla promotes the formation of an immunosuppressive niche and dampens antitumor surveillance. Consequently, elucidating the molecular circuitry linking Kla to macrophage immunobiology presents compelling opportunities for developing targeted antitumor strategies.

Within the TME, Kla reprograms macrophage polarization to drive oncogenesis. TAMs typically adopt an immunosuppressive M2 phenotype, with histone Kla levels directly correlating with this pro-tumorigenic shift. Mechanistically, Kla modification orchestrates TAM-dependent tumor progression through tissue-specific pathways: in gastric cancer, H3K18la induces sustained VCAM1 expression to activate AKT/mTOR/CXCL1 signaling and facilitate M2 macrophage recruitment (68); in prostate cancer, Kla inhibition restores the phagocytic capacity of macrophages to suppress tumor growth (69); in colorectal cancer, tumor-derived lactate promotes macrophage H3K18la to repress RARγ transcription and elevate IL-6, which activates STAT3/c-Myc signaling in cancer cells to reinforce M2-like TAM polarization (70); and in glioma, lactate-driven Kla remodels the immunosuppressive landscape by upregulating CD73 (tumor cells), CD39/CCR8 (Treg cells), CD39 (macrophages), and CD73 (T cells), establishing a purinergic cascade where CD39 hydrolyzes ATP to AMP followed by CD73-mediated conversion to adenosine to create an immunosuppressive niche (71). Concurrently, histone Kla in TAMs elevates IL-10 production, a cytokine that induces T-cell anergy through PD-1/LAG-3 upregulation (72). These findings position Kla as a key regulator of TAM-driven immune evasion.

Studies have shown that Kla modification sites exist in TAMs, and Kla levels correlate with macrophage transition to the M2 phenotype. Combination therapy with PI3K and MEK inhibitors inhibited histone Kla (H3K18la) in TAMs and controlled tumor growth in 80% of PTEN/p53-deficient prostate cancer mice. In the remaining mice that were non-responsive (20%), feedback activation of the Wnt/β-catenin pathway in the cerebral cortex led to recovery of H3K18la and inhibition of macrophage immune activity. Furthermore, adding a Wnt pathway inhibitor increased the remission rate to 100% in mice with prostate cancer (73). Therefore, targeting Kla modification may exert a positive antitumor effect by influencing these cells. In aggressive cancers (such as undifferentiated thyroid and pancreatic cancer), VSIG4-positive TAMS (VSIG4^+^ TAMs) regulate SPP1 through Kla modification, promoting neutrophil infiltration and impairing antigen-specific immunity, forming an immunosuppressive TME. The VSIG4 gene deletion reduces lactate and H3K18la production, thereby decreasing STAT3-mediated SPP1 transcription and disrupting intercellular interactions between TAMs and neutrophils, improving the immunosuppressive microenvironment (74).

**Figure 2:**
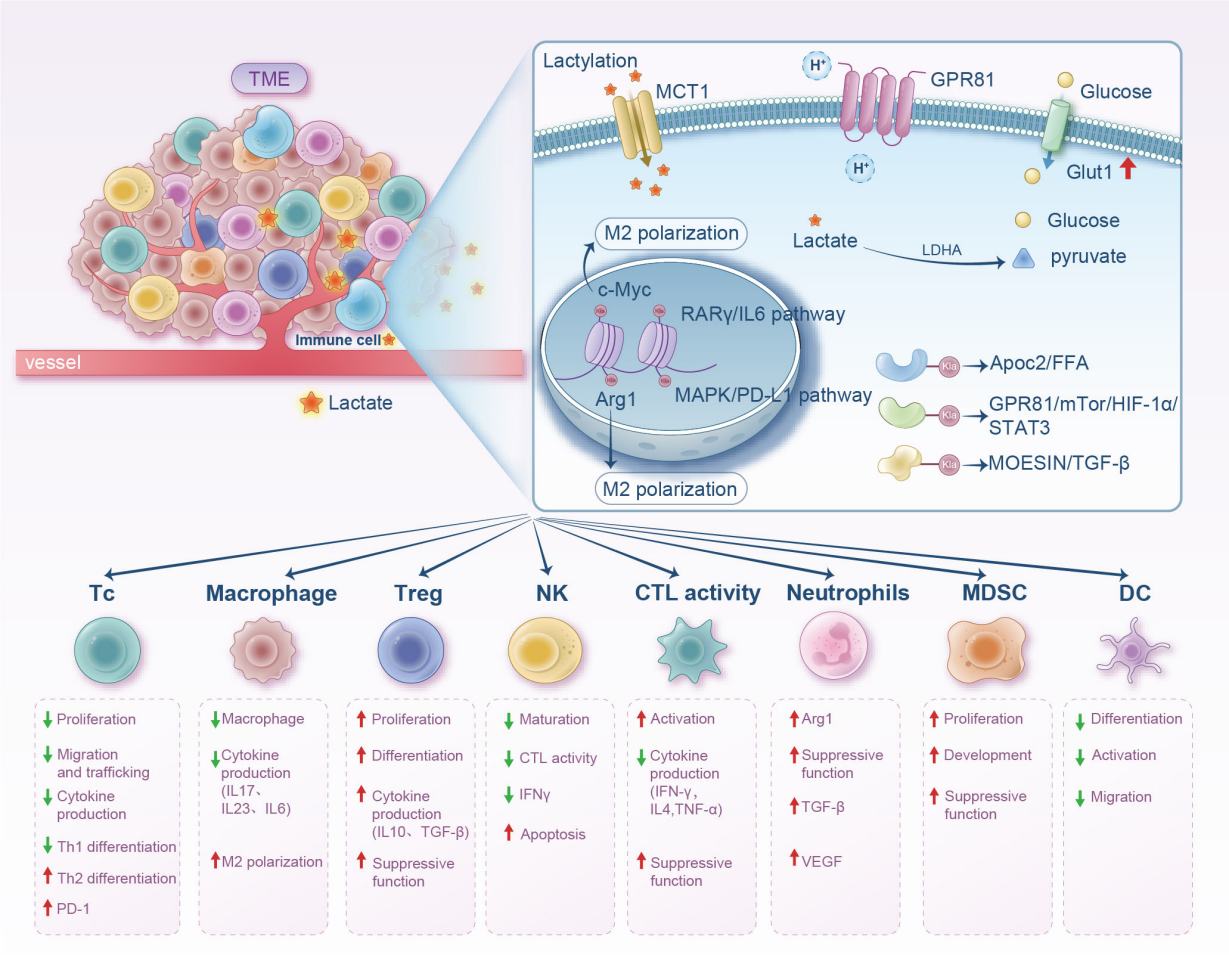
Diagram illustrating the impact of lactate within the tumor microenvironment on various immune cells. It shows lactate uptake via MCT1 and GPR81, leading to M2 polarization with related pathways and effects like cytokine production and cell differentiation. The lower section outlines immune cell types like T cells, macrophages, and dendritic cells with corresponding proliferation, differentiation, and suppressive functions influenced by lactate. Arrows indicate increases or decreases in activity or production.

TAMs are highly heterogenous in the TME, with different subsets regulating tumor progression and treatment resistance through epigenetic, metabolic, and signaling pathways. TAM biomarkers, such as CD68, CD163, and TREM2, among others, are associated with clinical outcomes in various solid tumors, whereas CD68 is associated with a good prognosis in colorectal cancer but typically reflects a poor outcome in other cancers (75). TAMs support tumor growth, metastasis, and drug resistance by secreting cytokines, promoting angiogenesis, and mediating immunosuppression, contributing substantially to, particularly virus-related cancers. Therapeutic strategies targeting TAMs (such as inhibiting TREM2 or SPP1) are potential interventions in clinical trials; nonetheless, the challenges of target specificity and potential side effects (such as metabolic disorders) need to be addressed (76) (**Figure 2**).

### 3.2 T cells

T cells are primarily divided into two subtypes: CD8^+^ and CD4^+^ T cells. CD4^+^ T cells are further classified into helper T lymphocytes (such as Th1, Th3, and TH17) and Tregs. CD8^+^ T cells, also known as CTLs, recognize specific antigens on tumor cell surfaces and directly kill tumor cells by releasing cytotoxic substances like perforin and granzyme. Correspondingly, CD4^+^ T cells mainly regulate the activity of other immune cells through cytokine secretion (77). For example, Th1 cells produce IFN-γ and IL-2, which play a crucial role in activating macrophages and enhancing T cell cytotoxicity (78). However, Treg cells inhibit immune responses by secreting IL-10 and TGF-β and can directly interact with effector T cells to suppress the activity of antitumor immune cells, aiding tumor cells in evading immune surveillance and attack (79).

Reportedly, lactate in the TME induces H3K18la modification, which regulates immune cell functions by enhancing the transcriptional activity of CD39, CD73, and CCR8 gene promoters. Specifically, upregulation of the CCR8 signaling pathway promotes the activation of Tregs, reinforcing immunosuppression in the TME and disrupting the dynamic balance between Th17 and Treg cells (80). Additionally, significant H3K18la enrichment is detected in Th3, Th1, and Treg cells, indicating that histone Kla is a common PTM in activated T cells (71). In another study, findings revealed that lactate in the TME induces H3K18la, increasing the activity of CD39, CD73, and CCR8 gene promoters. Moreover, CCR8 pathway upregulation activates Treg cells, enhancing immunosuppression and disrupting the Th17/Treg balance (81).

Notably, Kla modification plays a key role in the tumor immune microenvironment (TIME), reshaping immune cell functions, such as inhibiting the activity of T cells and NKT cells and enhancing the immunosuppressive function of Tregs. These effects are achieved by activating specific genes (such as *Arg1*, *PD-L1*) and signaling pathways (such as TGF-β), aiding tumor cells in evading immune surveillance (4, 82, 83). Reportedly, high lactate levels suppress the cytotoxicity and cytokine secretion of CD8^+^ T cells and promote the immunosuppressive function of Tregs, helping tumors evade immune surveillance. The acidic environment due to lactate accumulation in the TME directly inhibits the activation and proliferation of CD8^+^ T cells while supporting the survival and function of Tregs through metabolic reprogramming (such as enhanced oxidative phosphorylation), thus forming an immunosuppressive microenvironment (84, 85). Lactate further inhibits the antitumor activity of T cells by upregulating immune checkpoint molecules like PD-L1, providing an important metabolic basis for tumor immune escape through this multiple-action mechanism (86).

PD-L1 is an immune checkpoint protein frequently overexpressed in tumor cells across various solid malignancies, including lung cancer, melanoma, and gastric carcinoma. It mediates immune evasion by binding to the PD-1 receptor on T cells, suppressing T cell activation and effector functions (87). The PD-L1/PD-1 interaction inhibits critical T cell signaling pathways (e.g., PI3K/AKT/mTOR), ultimately leading to exhaustion and apoptosis of the T cells, creating an immunosuppressive “shield” for tumors (88, 89). The expression mechanisms of PD-L1 in tumors may involve signaling pathways within the TME, such as IFN-γ-induced PD-L1 expression, or activation of oncogenic signaling pathways, including PI3K/AKT and MAPK, to promote PD-L1 expression (90, 91). Additionally, epigenetic regulations such as histone modifications may also influence PD-L1 expression levels (82, 92). High PD-L1 expression is generally associated with poorer prognosis, though this may vary by tumor type. For example, in non-small cell lung cancer, high PD-L1 expression may reflect a better response to immunotherapy, while in other tumors, it may indicate stronger invasiveness (93).

Findings show that in acute myeloid leukemia, lactate, as a substrate, promotes the nuclear translocation of E3 binding protein (E3BP) through histone Kla modification (such as H3K18 and H4K5, among others). E3BP binds to the lactylated histone H4K5, enhancing the Kla level of the PD-L1 promoter region and activating PD-L1 transcription (92). Kla modification activates the promoter region of the PD-L1 gene, and this epigenetic regulation provides a molecular basis for high PD-L1 expression, further inhibiting the antitumor activity of CD8^+^ T cells (94, 95). Sun et al., in their study, identified the key role of Kla in the immunosuppressive microenvironment and treatment resistance of pancreatic ductal adenocarcinoma (PDAC), where high levels of Kla in PDAC are associated with an immunosuppressive TME, resulting in a reduction in CTLs (96). Huang et al. initially discovered that overall Kla levels are significantly elevated in colorectal cancer (CRC), especially in malignant tumors. Through tissue microarrays and *in vitro* experiments, they validated its correlation with tumor staging and poor prognosis, establishing Kla as an independent prognostic factor for CRC. Meanwhile, integrating single-cell transcriptome analysis (GSE132257) and The Cancer Genome Atlas (TCGA) data, they screened 23 Kla-related genes (LRGs) and constructed a prognostic risk model (LRGS), validating its predictive ability (AUC 0.7–0.8) in TCGA and Gene Expression Omnibus datasets. Patients in the high-risk group had significantly lower survival rates. Findings from further research showed that the TME of the high-risk group had reduced CD8^+^ T cells and increased expression of immune checkpoint genes (such as *PD-1*), increasing immune escape (97).

Reportedly, the glycolytic pathway is highly enriched in immune-escape tumors. Lactate upregulates B7-H3 expression through histone Kla modification (H3K18la), thereby inhibiting the antitumor immune activity of CD8^+^ T cells. It also promotes B7-H3 expression, reducing the infiltration ratio and cytotoxicity of CD8^+^ T cells in the TME, facilitating tumor progression. Inhibition of the glycolytic key enzyme LDHA or use of LDH inhibitors (such as sodium oxalate) can enhance CD8^+^ T cell killing ability, reverse tumor immune escape, and produce a synergistic effect with anti-PD-1 therapy. In animal models, targeting lactate metabolism (such as inhibiting LDHA or B7-H3) significantly suppresses tumor growth and activates tumor-infiltrating CD8^+^ T cells, providing a new strategy for combined immunotherapy (98) (**Figure 2**).

### 3.3 T-reg cells

Lactate accumulation induced by tumor cells suppresses effector T cells (which lack lactate-utilizing capacity) while supporting Treg function, enabling tumor cells to evade immune clearance. Watson et al. discovered the metabolic adaptation mechanism of Tregs in the TME, proposing that lactate is a key metabolic fuel for Tregs to maintain function in low-glucose and high-lactate environments. Tumor-infiltrating Treg cells (TIL-Tregs) exhibit reduced glucose uptake but enhanced lactate metabolism, a metabolic profile associated with stronger inhibitory function and stability (99). Tregs take up lactate via MCT1 (encoded by Slc16a1), convert it to pyruvate for entry into the TCA, and generate phosphoenolpyruvate (PEP) via PEP carboxykinase (PEPCK), which feeds back to promote gluconeogenesis and nucleotide synthesis to support Treg proliferation. Slc16a1 (MCT1) knockout preserves peripheral Treg function but significantly impairs intratumoral Treg inhibitory function, restricting tumor growth (99). This finding reveals the metabolic flexibility of Tregs in the TME, reducing glucose dependence and enhancing lactate metabolism to maintain immunosuppressive function. Additionally, MCT1 knockout synergizes with anti-PD-1 therapy to significantly prolong survival, suggesting that inhibiting MCT1 or TME acidification may disrupt Treg metabolic support and enhance the efficacy of immunotherapy. For example, MCT1 knockout combined with anti-PD-1 therapy significantly improves the remission rate in B16 melanoma. The metabolic adaptability of Tregs also highlights the complexity of tumor therapy, necessitating simultaneous intervention in metabolic limitations of effector T cells (e.g., glucose competition) and alternative metabolic pathways of Tregs (e.g., lactate utilization) (100).

Kla modification exerts dual regulatory effects on T cell-mediated immune responses in the TME. Lactate, a glycolytic metabolite and a regulator via Kla influences histone and non-histone functions to maintain a balance between immunosuppression and immune activation. High lactate levels suppress CD8^+^ T cell cytotoxicity and cytokine secretion while promoting Treg immunosuppressive function, aiding tumor immune evasion (60). *In vitro* experiments show that high lactate environments enhance Treg stability, whereas lactate degradation reduces their inductive effect (101). Specifically, high lactate levels increase Kla of moesin (a membrane-cytoskeleton linker protein) in these cells, with lactylated MOESIN enhancing TGF-β pathway efficacy to promote stability and generation of these immunosuppressive cells (83).

Additionally, intratumoral microbiota (e.g., *Escherichia coli*) promotes colorectal cancer liver metastasis by increasing lactate production. Lactate inhibits NF-κB signaling via Kla of RIG-I protein, leading to M2 macrophage polarization, which further suppresses Nlrp3 transcription, weakening CD8^+^ T cell antitumor activity and enhancing regulatory Treg immunosuppression. Moreover, lactate modulates immune cell phenotype and function via Kla (e.g., histone H3K18 lactylation), promoting Treg proliferation/suppression and inhibiting dendritic cell (DC) antigen presentation (60). In these mechanisms, an immunosuppressive TME is collectively constructed, where lactate further reinforces tumor immune escape by activating GPR81 receptor and regulating metabolic enzymes (e.g., p300) (102) (**Figure 2**).

### 3.4 NKT cells

NKT cells originate from the T cell lineage and express partial T cell markers; nevertheless, their unique TCR structure, antigen recognition mode (CD1d-dependent), and functional characteristics classify them as an independent innate-like lymphocyte subset (103). Despite not being traditional T cells, their “bridge” role (connecting innate and adaptive immunity) grants them a unique status in immune regulation, tumor surveillance, and disease pathogenesis, complementing classical T cells functionally (104). NKT cells are divided into two major subsets: type I NKT cells (the majority, with conserved T cell receptor (TCR) Vα24-Jα18, recognizing CD1d-presented glycolipid antigens like α-GalCer) and type II NKT cells (with diverse TCRs, recognizing self-lipid antigens, such as Lyso-GM1) (105). Activated type I NKT cells release perforin/granzyme to induce tumor cell apoptosis, express FasL to initiate apoptosis via binding to tumor cell Fas, and secrete cytokines (IFN-γ, IL-4, tumor necrotic factor-α) to enhance NK/CD8^+^ T cell cytotoxicity (106). They also induce DC maturation via IL-12/IL-18 secretion to promote antigen presentation and T cell activation, stimulating antitumor immunity (107). Type I NKT cells can suppress MDSC and Treg immunosuppression via IFN-γ secretion, while type II NKT cells antagonize type I NKT cells (105).

Previous studies show that extracellular low pH alone impairs NKT cell function, with the acidic microenvironment of the tumor potentially interfering with NKT cell function through metabolic control. Kla modification in the TME promotes immunosuppressive NKT cell generation. Under acidic TME conditions, FOXP3^+^ cells act as potent immunosuppressants, and prolonged lactate accumulation induces upregulation of H3K18 lactylation in NKT cells, leading to FOXP3^+^ NKT cell production (108). Moreover, Kla remodels immune cell functions (e.g., by activating genes like *Arg1/PD-L1* and pathways like TGF-β), inhibiting NKT cell activity to facilitate tumor immune evasion (109) (**Figure 2**).

### 3.5 NK cells

Harnessing innate immunity to expand antitumor responses is an attractive strategy. NK cells, specialized immune effector cells in the innate immune system, considerably influence tumor immune surveillance. Reduced NK cell activity correlates with increased cancer susceptibility and metastasis risk (110). Unlike T/NKT cells, NK cells lack clonotypic TCR and associated CD3 complex for signal transduction. However, endowed with potent cytotoxicity, NK cells exert strong responses by releasing cytolytic granules and cytotoxic cytokines after forming immune synapses with targets (111). In patients with cancer, these cells often exhibit dysfunctional phenotypes characterized by altered gene expression profiles and reduced cytotoxicity (112). Additionally, they are termed “immunoregulatory cells” for their ability to produce cytokines/chemokines that shape B/T cell responses and influence DC/macrophage/neutrophil functions, reflecting the complex biological network underlying their functions and supporting their value in immunotherapy (113, 114).

Reports reveal that lactate suppresses NK cell activation by inhibiting the expression of the activating receptor NKp46 (115). When lactate concentration in the TME exceeds 20 mM, it may induce NK cell apoptosis. Lactate also modulates immune escape by activating GPR81 on cell membranes, affecting NK cell function (116). These findings indicate that lactate directly or indirectly weakens NK cell-mediated tumor immune surveillance.

Wu et al. constructed a Cox risk model for hepatocellular carcinoma involving eight genes (e.g., *ARHGEF37, NR6A1*), finding that patients with high-risk scores had lower survival rates, with high-risk scores (correlated with Kla-related gene expression) negatively associated with low NK cell scores, suggesting Kla promotes tumor immune escape by inhibiting NK cell activity. Kla-related genes (e.g., *NR6A1, OSBP2, UNC119B*) that suppress NK cell function were further identified, with their expression linked to impaired immunotherapy response. This indicates these genes may enhance tumor immune escape by inhibiting NK cell function. Additionally, histone Kla induced by lactate accumulation is valuable for breast cancer (BC) prognosis. A Cox model based on Kla-specific genes (e.g., *CCR7, IGFBP6*) enables the use of risk scores as independent biomarkers. The significant correlation between Kla-specific genes and lactate accumulation-related genes (e.g., P300, LDHA) reveals that Kla promotes BC progression and immune escape by affecting NK cell function and multiple signaling pathways (NOTCH, WNT, among others) (117).

In another study, Kla modification was shown to affect NK cells in the TME mainly by regulating immune cell infiltration, shaping “cold” TMEs, and inhibiting immunoregulatory pathways. Pan-cancer analysis through TIMER2.0 and ImmuCellAI databases revealed a significant negative correlation between Kla score and NK cell infiltration, particularly in adrenocortical carcinoma, uterine carcinosarcoma, and endometrial carcinoma, among others. Kla suppresses NK cell function by creating “cold TMEs,” with high Kla scores correlating negatively with TME stromal/immune/estimation scores and positively with tumor purity, indicating promotion of “immune desert”-type TMEs where reduced NK cell infiltration weakens tumor immune surveillance (118). Gene Set Variation Analysis showed that Kla scores correlated negatively with immunoregulatory pathways (IL-6/JAK/STAT3 signaling, IFN-γ response, IL-2/STAT5 signaling, among others), whose inhibition may impair NK cell activation/proliferation/cytotoxicity. For example, downregulated IFN-γ response reduces NK cell recognition/killing efficiency against tumor cells. Patients with high Kla scores show poorer responses to immune checkpoint blockade (ICB) therapy with higher disease progression rates, as ICB efficacy correlates closely with TME immune cell infiltration. Reduced infiltration of NK cells and other immune cells due to high Kla may underlie immunotherapy resistance. Mutations/abnormal expression of Kla-related genes (*CREBBP, EP300, HDAC2*, among others) may affect NK cell-related gene transcription via epigenetic mechanisms. For instance, high mutation rates in CREBBP/EP300 may regulate NK cell activating receptor (e.g., NKG2D) expression (119, 120). As a substrate for Kla, lactate accumulation indirectly influences NK cell recruitment/function in the TME by driving histone/non-histone Kla (15). Future intervention strategies targeting the lactate-Kla axis (e.g., inhibiting lactate production or regulating Kla enzymes) may become novel approaches to enhance NK cell antitumor activity and overcome immunotherapy resistance (**Figure 2**).

### 3.6 Neutrophils

Neutrophils are a crucial component in the TME, exhibiting significant phenotypic and functional heterogeneity to either exert antitumor effects or promote tumor progression via multiple mechanisms. This bidirectional regulation is closely associated with their differentiation status and cytokine microenvironment in the TME (121, 122).

Studies have uncovered the molecular mechanism by which CD71^+^ neutrophils in the brain TME acquire immunosuppressive functions via hypoxia-induced metabolic reprogramming. Brain tumor-infiltrating neutrophils can be divided into functionally heterogeneous subsets based on CD71 expression, with the CD71^+^ subset displaying high glycolytic activity. Single-cell sequencing and ATP consumption experiments reveal that CD71^+^ neutrophils specifically accumulate in tumor hypoxic/glycolytic regions. Hypoxia enhances glycolytic flux by upregulating GLUT1/LDHA, and intracellular lactate accumulation triggers p300-induced histone H3K18 lactylation modification, directly regulating arginase-1 (ARG1) expression to suppress CD8^+^/CD4^+^ T cell activity and promote tumor immune escape. This modification specifically binds to the ARG1 gene promoter to drive its expression, a process significantly inhibited by GNE-140 (LDHA inhibitor) or isosafrole (123).

Lactate also regulates other immune cells in the TME, such as MDSCs and DCs. MDSCs, a heterogeneous population derived from immature bone marrow, serve as precursors of mature monocytes/DCs/granulocytes and are key cells promoting tumor proliferation and immune suppression (124). Lactate induces MDSC accumulation via granulocyte-macrophage colony-stimulating factor and IL-6 (125). Additionally, MDSCs are essential for TAM accumulation. When these cells migrate to tumor sites, hypoxia and local acidification increase CD45 phosphatase activity, promoting their conversion to TAMs (126). Lactate-induced HIF1-α also promotes MDSC differentiation into TAMs by regulating inducible nitric oxide synthase and ARG1 expression, contributing to adaptive immune suppression (127). In a study on pancreatic cancer radiotherapy, MDSC immunosuppressive phenotypes were enhanced by lactate via the GPR81/mTOR/HIF-1α/STAT3 pathway (128) (**Figure 2**). Lactate suppresses T cell-mediated immune responses by activating the HIF-1α/NDUFA4L2 signaling pathway in DCs and controls immune escape via paracrine activation of GPR81 on DCs (129) (**Figure 2**). Therefore, targeting lactate metabolism may regulate MDSC/DC-mediated immunosuppression.

## 4 Lactylation in tumor immunotherapy

With the continuous in-depth research into lactate and Kla mechanisms in the TME, a growing interest exists in the impact of Kla on tumors and therapeutic responses. Histone Kla is sensitive to lactate levels: inhibiting glycolysis impairs lactate production, subsequently reducing Kla, while increased lactate production elevates Kla levels (130). Patients with breast cancer have undergone histone Kla-related gene analyses, identifying Kla-related targets to guide immunotherapy for related tumors (131). As in breast cancer, oral cancer tissues also induced mesenchymal markers and cancer cell migration after L - lactate treatment, but these changes were significantly neutralized by the mitochondrial pyruvate carrier inhibitor 7ACC2 (132). Oxalate, which inhibits lactate production, downregulates CD39/CD73/CCR8 gene promoter activity by reducing histone H3K18 lactylation. It also modifies the immunosuppressive TME, promoting immune activation, suggesting that enhancing chimeric antigen receptor cell function may represent a potential strategy for glioblastoma therapy (71). In a non-small cell lung cancer study, Kla of apolipoprotein C-2 (APOC2) at K70 promotes Treg enhancement and immunotherapy resistance by inducing elevated free fatty acid levels (101). A specific antibody against the APOC2 K70 lactylation site has been developed, showing a positive correlation with immunotherapy resistance in non-small cell lung cancer (101). These findings reveal the feasibility of combination therapy via developing site-specific antibodies against Kla. However, no therapies targeting histone Kla site-specific antibodies have been studied, possibly because of challenges in blocking histone Kla without affecting other normal cell functions. The effects of various histone Kla inhibitors on other cellular functions remain unclear.

**Figure 3:**
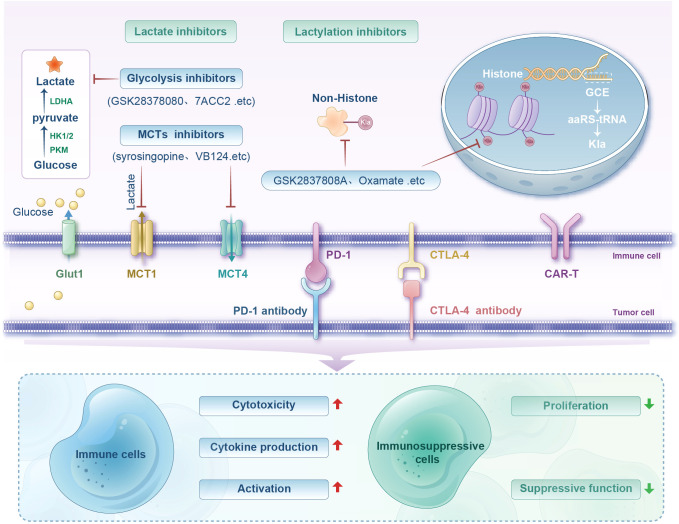
Diagram illustrating lactate and lactylation inhibitors' roles in immune regulation. Shows processes involving glucose, lactate, and inhibitors like GSK2837808A and Oxamate. Includes pathways for immune cell activation, cytotoxicity, and cytokine production, and their influence on immunosuppressive cell proliferation and function.

Lactate is a key substrate involved in tumor immunosuppressive microenvironment formation and a necessary condition for protein Kla at high concentrations. Kla is sensitive to lactate levels: glycolysis inhibition reduces lactate production and subsequently decreases histone Kla, whereas increased lactate production raises Kla levels (133). Lactate production- and transport-related enzymes, such as MCTs and LDHA, have long been considered in antitumor immunotherapy. MCT1, proven as a key regulator of lactate exchange between tumor cells, serves as an HIF-1α inhibitor when blocked (134). Given its critical role in regulating glycolysis, MCT1 inhibition is an established therapeutic modality with antimetabolic activity. A preclinical study showed that combining the MCT1 inhibitor AZD3965 with anti-PD-1 therapy reduces lactate release into the TME, enhancing tumor immune efficacy (48). Additionally, combining anti-PD-1 with LDH inhibitors exhibits stronger antitumor effects than using anti-PD-1 alone (135). Inhibiting MCT4 reverses lactate-induced immunosuppression, and blocking MCT4 improves the efficacy of *in vivo* immune checkpoint blockade (136). In another study of hepatocellular carcinoma (HCC), inhibition of MCT4 by using VB124 enhanced CD8^+^ T-cell infiltration and cytotoxicity, thereby inhibiting tumor growth. It is also noteworthy that the combination of MCT4 inhibitors with anti-PD-1 immunotherapy significantly improved the outcome of HCC patients (137). Furthermore, *in vitro* intervention of lactate in Naïve CD4^+^ T cells can promote the differentiation of CD4^+^ T cells into Treg by increasing mTOR phosphorylation and HIF-1α synthesis, while the addition of lactate uptake inhibitor AZD3965, LDHA inhibitor GSK2837808A, and NADH conversion inhibitor Rotenone can reverse this differentiation (138). Therefore, inhibiting endogenous lactate production and transport-related enzymes (e.g., MCTs, LDHA) can be applied to tumor therapy (**Figure 3**).

Intriguingly, Zong et al. proposed a method to introduce Kla at specific protein sites via genetic code expansion (GCE) technology. Compared with those of traditional PTM research methods (enzymatic regulation, chemical synthesis, or site-directed mutagenesis), GCE technology avoids imprecision caused by the broad substrate specificity of enzymes and the inability of mutations to fully replicate natural modification functions. GCE enables efficient suppression of amber codons in bacteria and mammalian cells with the use of orthogonal aminoacyl-tRNA synthetase-tRNA pairs, achieving site-specific introduction of Kla into target proteins. This approach avoids the ambiguity of mutational or enzymatic methods and ensures minimal disruption to natural cellular mechanisms, making experiments reliable and physiologically relevant. Furthermore, Zong et al. successfully introduced lactylation groups into two lysine residues of the p53 DNA-binding domain, systematically investigating their functional effects on p53 activity *in vitro* and *in vivo*. They demonstrated the versatility of GCE technology in exploring PTMs, particularly the diverse Kla effects, and provided a powerful tool for revealing new regulatory mechanisms and interactions. Additionally, GCE technology combined with ultrasensitive proteomics could further illuminate Kla regulation in the tumor immune suppression system and advance synthetic biology and precision medicine (139) (**Figure 3**).

In summary, histones and non-histones of tumor-related immune cells harbor abundant Kla sites. Exploring the mechanisms and regulatory sites of Kla can help to gradually identify safer and more effective therapeutic targets for antitumor immunotherapy, uncovering new directions for combination immunotherapy strategies. Moreover, reducing tumor lactate levels inhibits Kla and disrupts the lactate metabolic balance in the TME, representing a promising cancer treatment option that has already been implemented in several preclinical and clinical trials. Therefore, it is also necessary to establish synergistic effects between lactate inhibitors and other adjuvant therapies.

In conclusion, lactate plays a key role in the tumor immunosuppressive microenvironment and the mechanisms by which high lactate levels affect immune cells through Kla warrant further investigation. The focus of most emerging therapeutic strategies currently is on developing glycolysis and lactate inhibitors, with few interventions directly targeting Kla pathways, leaving significant room for exploration. Balanced regulation of the dual effects of lactate and Kla may provide new insights for overcoming tumor-induced immunosuppression and potentially enhance the efficacy of cancer immunotherapies (**Figure 3**).

## 5 Conclusion and perspectives

Although an expanding corpus of research indicates that lactylation modulates transcriptional programs within TME immune cells to facilitate oncogenesis, significant challenges persist. First, while preliminary investigations have probed the interplay among immune cell Kla, lactate-driven metabolism, competing post-translational modifications (*e.g.*, acetylation, ubiquitination), and immunosuppressive microenvironment dynamics, the mechanistic underpinnings remain inadequately elucidated. Second, precise mapping of Kla sites constitutes a critical bottleneck in deciphering its molecular pathophysiology. Current literature predominantly explores how tumor cell Kla governs biological functions within the TME, whereas site-directed mutagenesis approaches to validate specific Kla residues remain conspicuously underreported. Moreover, conventional analytical methodologies like tandem mass spectrometry, despite their precision, suffer from operational complexity and protracted analytical timelines.

Concurrently, technological revolutions are reshaping the investigative landscape. Large-scale sequencing, machine learning, and multi-omics integration have profoundly advanced our understanding of immune cell heterogeneity, metabolic reprogramming, and Kla site identification within the TME. These advances similarly accelerate targeted therapeutic development. Isotopic tracing coupled with super-resolution imaging now permits real-time tracking of Kla spatiotemporal dynamics in living systems, revealing its metabolic regulation and subcellular distribution. Complementarily, chromatin immunoprecipitation sequencing enables genome-wide mapping of Kla-enriched domains. The synergistic application of these orthogonal methodologies affords unprecedented insights into Kla’s physiological and pathological roles across cellular contexts.

Computational biology has further catalyzed progress: predictive algorithms (*e.g.*, FSL-Kla, Auto-Kla) now expedite high-fidelity Kla target identification. Integrative multi-omics analytics and artificial intelligence-guided machine learning are positioned to unlock transformative discoveries in Kla research. Seminal studies demonstrate that targeting histone Kla in immune cells can restore effector functions and remodel immunosuppressive niches, establishing this axis as a therapeutically compelling frontier. Notably, structure-based drug design targeting novel lactylation sites—particularly when integrated with established immunotherapies such as checkpoint inhibitors (anti-PD-1/anti-CTLA-4 antibodies) and CAR-T—constitutes a rationally prioritized therapeutic strategy to augment antitumor responses, representing a critical frontier warranting systematic interrogation.

## References

[1] Fu Q Cat A Zheng YG. New histone lysine acylation biomarkers and their roles in epigenetic regulation. Curr Protoc. (2023) 3:e746. doi: 10.1002/cpz1.746, PMID: 37098732 PMC13528283

[2] Liu R Wu J Guo H Yao W Li S Lu Y. Post-translational modifications of histones: Mechanisms, biological functions, and therapeutic targets. MedComm. (2023) 4:e292. doi: 10.1002/mco2.292, PMID: 37220590 PMC10200003

[3] Xu H Wu M Ma X Huang W Xu Y. Function and mechanism of novel histone posttranslational modifications in health and disease. BioMed Res Int. (2021) 2021:6635225. doi: 10.1155/2021/6635225, PMID: 33763479 PMC7952163

[4] Zhang D Tang Z Huang H Zhou G Cui C Weng Y. Metabolic regulation of gene expression by histone lactylation. Nature. (2019) 574:575–80. doi: 10.1038/s41586-019-1678-1, PMID: 31645732 PMC6818755

[5] Zhang C Zhou T Li C Wang D Tao J Zhu X. Deciphering novel enzymatic and non-enzymatic lysine lactylation in Salmonella. Emerg Microbes Infect. (2025) 14:2475838. doi: 10.1080/22221751.2025.2475838, PMID: 40035788 PMC11924271

[6] Yu X Yang J Xu J Pan H Wang W Yu X. Histone lactylation: from tumor lactate metabolism to epigenetic regulation. Int J Biol Sci. (2024) 20:1833–54. doi: 10.7150/ijbs.91492, PMID: 38481814 PMC10929197

[7] Zhu Z Zheng X Zhao P Chen C Xu G Ke X. Potential of lactylation as a therapeutic target in cancer treatment (Review). Mol Med Rep. (2025) 31:91. doi: 10.3892/mmr.2025.13456, PMID: 39950331 PMC11836599

[8] Sun L Zhang H Gao P. Metabolic reprogramming and epigenetic modifications on the path to cancer. Protein Cell. (2022) 13:877–919. doi: 10.1007/s13238-021-00846-7, PMID: 34050894 PMC9243210

[9] Kocianova E Piatrikova V Golias T. Revisiting the warburg effect with focus on lactate. Cancers. (2022) 14:6028. doi: 10.3390/cancers14246028, PMID: 36551514 PMC9776395

[10] Chen J Huang Z Chen Y Tian H Chai P Shen Y. Lactate and lactylation in cancer. Signal Transduct Target Ther. (2025) 10:38. doi: 10.1038/s41392-024-02082-x, PMID: 39934144 PMC11814237

[11] Luo Y Zhang N Ye J Wang Z Zhou X Liu J. Unveiling lactylation modification: A new hope for cancer treatment. BioMed Pharmacother. (2025) 184:117934. doi: 10.1016/j.biopha.2025.117934, PMID: 39986235

[12] Sharma N Fan X Atolagbe OT Ge Z Dao KN Sharma P. ICOS costimulation in combination with CTLA-4 blockade remodels tumor-associated macrophages toward an antitumor phenotype. J Exp Med. (2024) 221:e20231263. doi: 10.1084/jem.20231263, PMID: 38517331 PMC10959121

[13] Du Q Meng C Zhang W Huang L Xue C. Establishing a prognostic model correlates to inflammatory response pathways for prostate cancer via multiomic analysis of lactylation-related genes. Int J Genomics. (2025) 2025:6681711. doi: 10.1155/ijog/6681711, PMID: 40161494 PMC11952923

[14] Chen L Huang L Gu Y Cang W Sun P Xiang Y. Lactate-lactylation hands between metabolic reprogramming and immunosuppression. Int J Mol Sci. (2022) 23:11943. doi: 10.3390/ijms231911943, PMID: 36233246 PMC9569569

[15] Jin M Cao W Chen B Xiong M Cao G. Tumor-derived lactate creates a favorable niche for tumor via supplying energy source for tumor and modulating the tumor microenvironment. Front Cell Dev Biol. (2022) 10:808859. doi: 10.3389/fcell.2022.808859, PMID: 35646923 PMC9136137

[16] Garofano F Schmidt-Wolf IGH. High expression of cannabinoid receptor 2 on cytokine-induced killer cells and multiple myeloma cells. Int J Mol Sci. (2020) 21:3800. doi: 10.3390/ijms21113800, PMID: 32471216 PMC7312510

[17] Wang L Li S Luo H Lu Q Yu S. PCSK9 promotes the progression and metastasis of colon cancer cells through regulation of EMT and PI3K/AKT signaling in tumor cells and phenotypic polarization of macrophages. J Exp Clin Cancer Res. (2022) 41:303. doi: 10.1186/s13046-022-02477-0, PMID: 36242053 PMC9563506

[18] Ding C-H Yan F-Z Xu B-N Qian H Hong X-L Liu S-Q. PRMT3 drives PD-L1-mediated immune escape through activating PDHK1-regulated glycolysis in hepatocellular carcinoma. Cell Death Dis. (2025) 16:158. doi: 10.1038/s41419-025-07482-7, PMID: 40050608 PMC11885674

[19] Deng X Huang Y Zhang J Chen Y Jiang F Zhang Z. Histone lactylation regulates PRKN-Mediated mitophagy to promote M2 Macrophage polarization in bladder cancer. Int Immunopharmacol. (2025) 148:114119. doi: 10.1016/j.intimp.2025.114119, PMID: 39854875

[20] Su J Zheng Z Bian C Chang S Bao J Yu H. Functions and mechanisms of lactylation in carcinogenesis and immunosuppression. Front Immunol. (2023) 14:1253064. doi: 10.3389/fimmu.2023.1253064, PMID: 37646027 PMC10461103

[21] Copsel SN Lightbourn CO Barreras H Lohse I Wolf D Bader CS. BET bromodomain inhibitors which permit treg function enable a combinatorial strategy to suppress GVHD in pre-clinical allogeneic HSCT. Front Immunol. (2018) 9:3104. doi: 10.3389/fimmu.2018.03104, PMID: 30733722 PMC6353853

[22] Chaudagar K Hieromnimon HM Khurana R Labadie B Hirz T Mei S. Reversal of lactate and PD-1–mediated macrophage immunosuppression controls growth of PTEN/p53-deficient prostate cancer. Clin Cancer Res. (2023) 29:1952–68. doi: 10.1158/1078-0432.CCR-22-3350, PMID: 36862086 PMC10192075

[23] Rui R Zhou L He S. Cancer immunotherapies: advances and bottlenecks. Front Immunol. (2023) 14:1212476. doi: 10.3389/fimmu.2023.1212476, PMID: 37691932 PMC10484345

[24] Rabinowitz JD Enerbäck S. Lactate: the ugly duckling of energy metabolism. Nat Metab. (2020) 2:566–71. doi: 10.1038/s42255-020-0243-4, PMID: 32694798 PMC7983055

[25] Guyon J Fernandez-Moncada I Larrieu CM Bouchez CL Pagano Zottola AC Galvis J. Lactate dehydrogenases promote glioblastoma growth and invasion via a metabolic symbiosis. EMBO Mol Med. (2022) 14:e15343. doi: 10.15252/emmm.202115343, PMID: 36278433 PMC9728051

[26] Du M Yu T Zhan Q Li H Zou Y Geng M. Development of a novel lactate dehydrogenase A inhibitor with potent antitumor activity and immune activation. Cancer Sci. (2022) 113:2974–85. doi: 10.1111/cas.15468, PMID: 35722994 PMC9459323

[27] Massari F Ciccarese C Santoni M Iacovelli R Mazzucchelli R Piva F. Metabolic phenotype of bladder cancer. Cancer Treat Rev. (2016) 45:46–57. doi: 10.1016/j.ctrv.2016.03.005, PMID: 26975021

[28] Fontana F Giannitti G Marchesi S Limonta P. The PI3K/akt pathway and glucose metabolism: A dangerous liaison in cancer. Int J Biol Sci. (2024) 20:3113–25. doi: 10.7150/ijbs.89942, PMID: 38904014 PMC11186371

[29] Vaupel P Multhoff G. Revisiting the Warburg effect: historical dogma versus current understanding. J Physiol. (2021) 599:1745–57. doi: 10.1113/JP278810, PMID: 33347611

[30] Kasprzak A. Insulin-like growth factor 1 (IGF-1) signaling in glucose metabolism in colorectal cancer. Int J Mol Sci. (2021) 22:6434. doi: 10.3390/ijms22126434, PMID: 34208601 PMC8234711

[31] Li Y Zhang R Hei H. Advances in post-translational modifications of proteins and cancer immunotherapy. Front Immunol. (2023) 14:1229397. doi: 10.3389/fimmu.2023.1229397, PMID: 37675097 PMC10477431

[32] Zhang Y Xu Y Lu W Ghergurovich JM Guo L Blair IA. Upregulation of antioxidant capacity and nucleotide precursor availability suffices for oncogenic transformation. Cell Metab. (2021) 33:94–109.e8. doi: 10.1016/j.cmet.2020.10.002, PMID: 33159852 PMC7846267

[33] Kong W He J Zhou Q Zhou X Wei X Yang Y. Histone lactylation-related genes correlate with the molecular patterns and functions of cancer-associated fibroblasts and have significant clinical implications in clear cell renal cell carcinoma. Heliyon. (2024) 10:e33554. doi: 10.1016/j.heliyon.2024.e33554, PMID: 39035489 PMC11259888

[34] Kooshki L Mahdavi P Fakhri S Akkol EK Khan H. Targeting lactate metabolism and glycolytic pathways in the tumor microenvironment by natural products: A promising strategy in combating cancer. BioFactors Oxf Engl. (2022) 48:359–83. doi: 10.1002/biof.1799, PMID: 34724274

[35] Praharaj M Shen F Lee AJ Zhao L Nirschl TR Theodros D. Metabolic reprogramming of tumor-associated macrophages using glutamine antagonist JHU083 drives tumor immunity in myeloid-rich prostate and bladder cancers. Cancer Immunol Res. (2024) 12:854–75. doi: 10.1158/2326-6066.CIR-23-1105, PMID: 38701369 PMC11217738

[36] Chen J Zhang M Liu Y Zhao S Wang Y Wang M. Histone lactylation driven by mROS-mediated glycolytic shift promotes hypoxic pulmonary hypertension. J Mol Cell Biol. (2023) 14:mjac073. doi: 10.1093/jmcb/mjac073, PMID: 36564027 PMC10175659

[37] Chen J Wu F Cao Y Xing Y Liu Q Zhao Z. The novel role of LDHA/LDHB in the prognostic value and tumor-immune infiltration in clear cell renal cell carcinoma. PeerJ. (2023) 11:e15749. doi: 10.7717/peerj.15749, PMID: 37547725 PMC10402698

[38] Li J Ma P Liu Z Xie J--. and D-Lactate: unveiling their hidden functions in disease and health. Cell Commun Signal. (2025) 23:134. doi: 10.1186/s12964-025-02132-z, PMID: 40075490 PMC11905701

[39] Wang T Ye Z Li Z Jing D Fan G Liu M. Lactate-induced protein lactylation: A bridge between epigenetics and metabolic reprogramming in cancer. Cell Prolif. (2023) 56:e13478. doi: 10.1111/cpr.13478, PMID: 37060186 PMC10542650

[40] Jiang J Huang D Jiang Y Hou J Tian M Li J. Lactate modulates cellular metabolism through histone lactylation-mediated gene expression in non-small cell lung cancer. Front Oncol. (2021) 11:647559. doi: 10.3389/fonc.2021.647559, PMID: 34150616 PMC8208031

[41] Amrutkar M Berg K Balto A Skilbrei MG Finstadsveen AV Aasrum M. Pancreatic stellate cell-induced gemcitabine resistance in pancreatic cancer is associated with LDHA- and MCT4-mediated enhanced glycolysis. Cancer Cell Int. (2023) 23:9. doi: 10.1186/s12935-023-02852-7, PMID: 36658582 PMC9850604

[42] Zhang T Chen L Kueth G Shao E Wang X Ha T. Lactate’s impact on immune cells in sepsis: unraveling the complex interplay. Front Immunol. (2024) 15:1483400. doi: 10.3389/fimmu.2024.1483400, PMID: 39372401 PMC11449721

[43] Zheng P Mao Z Luo M Zhou L Wang L Liu H. Comprehensive bioinformatics analysis of the solute carrier family and preliminary exploration of SLC25A29 in lung adenocarcinoma. Cancer Cell Int. (2023) 23:222. doi: 10.1186/s12935-023-03082-7, PMID: 37775731 PMC10543265

[44] Wu D Kraut JA. Potential role of NHE1 (sodium-hydrogen exchanger 1) in the cellular dysfunction of lactic acidosis: implications for treatment. Am J Kidney Dis Off J Natl Kidney Found. (2011) 57:781–7. doi: 10.1053/j.ajkd.2010.10.058, PMID: 21349616

[45] Ward C Meehan J Gray ME Murray AF Argyle DJ Kunkler IH. The impact of tumour pH on cancer progression: strategies for clinical intervention. Explor Target Anti-Tumor Ther. (2020) 1:71–100. doi: 10.37349/etat.2020.00005, PMID: 36046070 PMC9400736

[46] Brooks GA Osmond AD Arevalo JA Duong JJ Curl CC Moreno-Santillan DD. Lactate as a myokine and exerkine: drivers and signals of physiology and metabolism. J Appl Physiol. (2023) 134:529–48. doi: 10.1152/japplphysiol.00497.2022, PMID: 36633863 PMC9970662

[47] Ye L Jiang Y Zhang M. Crosstalk between glucose metabolism, lactate production and immune response modulation. Cytokine Growth Factor Rev. (2022) 68:81–92. doi: 10.1016/j.cytogfr.2022.11.001, PMID: 36376165

[48] Huang T Feng Q Wang Z Li W Sun Z Wilhelm J. Tumor-targeted inhibition of monocarboxylate transporter 1 improves T-cell immunotherapy of solid tumors. Adv Healthc Mater. (2021) 10:e2000549. doi: 10.1002/adhm.202000549, PMID: 32431046 PMC7674253

[49] Yin D Jiang N Cheng C Sang X Feng Y Chen R. Protein lactylation and metabolic regulation of the zoonotic parasite toxoplasma gondii. Genomics Proteomics Bioinf. (2023) 21:1163–81. doi: 10.1016/j.gpb.2022.09.010, PMID: 36216028 PMC11082259

[50] Xie Y Hu H Liu M Zhou T Cheng X Huang W. The role and mechanism of histone lactylation in health and diseases. Front Genet. (2022) 13:949252. doi: 10.3389/fgene.2022.949252, PMID: 36081996 PMC9445422

[51] He Y Song T Ning J Wang Z Yin Z Jiang P. Lactylation in cancer: Mechanisms in tumour biology and therapeutic potentials. Clin Transl Med. (2024) 14:e70070. doi: 10.1002/ctm2.70070, PMID: 39456119 PMC11511673

[52] Xu Y Wan W. Acetylation in the regulation of autophagy. Autophagy. (2023) 19:379–87. doi: 10.1080/15548627.2022.2062112, PMID: 35435793 PMC9851266

[53] Sankar A Mohammad F Sundaramurthy AK Wang H Lerdrup M Tatar T. Histone editing elucidates the functional roles of H3K27 methylation and acetylation in mammals. Nat Genet. (2022) 54:754–60. doi: 10.1038/s41588-022-01091-2, PMID: 35668298

[54] Sun L Zhang Y Yang B Sun S Zhang P Luo Z. Lactylation of METTL16 promotes cuproptosis via m6A-modification on FDX1 mRNA in gastric cancer. Nat Commun. (2023) 14:6523. doi: 10.1038/s41467-023-42025-8, PMID: 37863889 PMC10589265

[55] Zhang D Gao J Zhu Z Mao Q Xu Z Singh PK. Lysine l-lactylation is the dominant lactylation isomer induced by glycolysis. Nat Chem Biol. (2025) 21:91–9. doi: 10.1038/s41589-024-01680-8, PMID: 39030363 PMC11666458

[56] Yang D Yin J Shan L Yi X Zhang W Ding Y. Identification of lysine-lactylated substrates in gastric cancer cells. iScience. (2022) 25:104630. doi: 10.1016/j.isci.2022.104630, PMID: 35800753 PMC9253728

[57] Li X Cai P Tang X Wu Y Zhang Y Rong X. Lactylation modification in cardiometabolic disorders: function and mechanism. Metabolites. (2024) 14:217. doi: 10.3390/metabo14040217, PMID: 38668345 PMC11052226

[58] Zhao Q Wang Q Yao Q Yang Z Li W Cheng X. Nonenzymatic lysine d-lactylation induced by glyoxalase II substrate SLG dampens inflammatory immune responses. Cell Res. (2025) 35:97–116. doi: 10.1038/s41422-024-01060-w, PMID: 39757301 PMC11770101

[59] Zhang C Zhou L Zhang M Du Y Li C Ren H. H3K18 lactylation potentiates immune escape of non–small cell lung cancer. Cancer Res. (2024) 84:3589–601. doi: 10.1158/0008-5472.CAN-23-3513, PMID: 39137401

[60] Gu J Xu X Li X Yue L Zhu X Chen Q. Tumor-resident microbiota contributes to colorectal cancer liver metastasis by lactylation and immune modulation. Oncogene. (2024) 43:2389–404. doi: 10.1038/s41388-024-03080-7, PMID: 38890429 PMC11281901

[61] Wang S Huang T Wu Q Yuan H Wu X Yuan F. Lactate reprograms glioblastoma immunity through CBX3-regulated histone lactylation. J Clin Invest. (2024) 134:e176851. doi: 10.1172/JCI176851, PMID: 39545414 PMC11563687

[62] Zhu R Ye X Lu X Xiao L Yuan M Zhao H. ACSS2 acts as a lactyl-CoA synthetase and couples KAT2A to function as a lactyltransferase for histone lactylation and tumor immune evasion. Cell Metab. (2025) 37:361–376.e7. doi: 10.1016/j.cmet.2024.10.015, PMID: 39561764

[63] Rho H Hay N. Protein lactylation in cancer: mechanisms and potential therapeutic implications. Exp Mol Med. (2025) 57:545–53. doi: 10.1038/s12276-025-01410-7, PMID: 40128358 PMC11958728

[64] Chen L Huang L Gu Y Li C Sun P Xiang Y. Novel post-translational modifications of protein by metabolites with immune responses and immune-related molecules in cancer immunotherapy. Int J Biol Macromol. (2024) 277:133883. doi: 10.1016/j.ijbiomac.2024.133883, PMID: 39033895

[65] Tan S Day D Nicholls SJ Segelov E. Immune checkpoint inhibitor therapy in oncology: current uses and future directions: JACC: cardioOncology state-of-the-art review. JACC CardioOncology. (2022) 4:579–97. doi: 10.1016/j.jaccao.2022.09.004, PMID: 36636451 PMC9830229

[66] Yunna C Mengru H Lei W Weidong C. Macrophage M1/M2 polarization. Eur J Pharmacol. (2020) 877:173090. doi: 10.1016/j.ejphar.2020.173090, PMID: 32234529

[67] Susser LI Nguyen M-A Geoffrion M Emerton C Ouimet M Khacho M. Mitochondrial fragmentation promotes inflammation resolution responses in macrophages via histone lactylation. Mol Cell Biol. (2023) 43:531–46. doi: 10.1080/10985549.2023.2253131, PMID: 37807652 PMC10569354

[68] Zhao Y Jiang J Zhou P Deng K Liu Z Yang M. H3K18 lactylation-mediated VCAM1 expression promotes gastric cancer progression and metastasis via AKT-mTOR-CXCL1 axis. Biochem Pharmacol. (2024) 222:116120. doi: 10.1016/j.bcp.2024.116120, PMID: 38461905

[69] Pan J Zhang J Lin J Cai Y Zhao Z. Constructing lactylation-related genes prognostic model to effectively predict the disease-free survival and treatment responsiveness in prostate cancer based on machine learning. Front Genet. (2024) 15:1343140. doi: 10.3389/fgene.2024.1343140, PMID: 38566813 PMC10985269

[70] Li X-M Yang Y Jiang F-Q Hu G Wan S Yan W-Y. Histone lactylation inhibits RARγ expression in macrophages to promote colorectal tumorigenesis through activation of TRAF6-IL-6-STAT3 signaling. Cell Rep. (2024) 43:113688. doi: 10.1016/j.celrep.2024.113688, PMID: 38245869

[71] Sun T Liu B Li Y Wu J Cao Y Yang S. Oxamate enhances the efficacy of CAR-T therapy against glioblastoma via suppressing ectonucleotidases and CCR8 lactylation. J Exp Clin Cancer Res CR. (2023) 42:253. doi: 10.1186/s13046-023-02815-w, PMID: 37770937 PMC10540361

[72] Ruan J Ouyang M Zhang W Luo Y Zhou D. The effect of PD-1 expression on tumor-associated macrophage in T cell lymphoma. Clin Transl Oncol Off Publ Fed Span Oncol Soc Natl Cancer Inst Mex. (2021) 23:1134–41. doi: 10.1007/s12094-020-02499-0, PMID: 33211280

[73] Chaudagar K Hieromnimon HM Kelley A Labadie B Shafran J Rameshbabu S. Suppression of tumor cell lactate-generating signaling pathways eradicates murine PTEN/p53-deficient aggressive-variant prostate cancer via macrophage phagocytosis. Clin Cancer Res. (2023) 29:4930–40. doi: 10.1158/1078-0432.CCR-23-1441, PMID: 37721526 PMC10841690

[74] Pan Z Chen J Xu T Cai A Han B Li Y. VSIG4+ tumor-associated macrophages mediate neutrophil infiltration and impair antigen-specific immunity in aggressive cancers through epigenetic regulation of SPP1. J Exp Clin Cancer Res. (2025) 44:45. doi: 10.1186/s13046-025-03303-z, PMID: 39920772 PMC11803937

[75] Wang J Yang P Yu T Gao M Liu D Zhang J. Lactylation of PKM2 suppresses inflammatory metabolic adaptation in pro-inflammatory macrophages. Int J Biol Sci. (2022) 18:6210–25. doi: 10.7150/ijbs.75434, PMID: 36439872 PMC9682528

[76] Kzhyshkowska J Shen J Larionova I. Targeting of TAMs: can we be more clever than cancer cells? Cell Mol Immunol. (2024) 21:1376–409. doi: 10.1038/s41423-024-01232-z, PMID: 39516356 PMC11607358

[77] Hao Z-N Tan X-P Zhang Q Li J Xia R Ma Z. Lactate and lactylation: dual regulators of T-cell-mediated tumor immunity and immunotherapy. Biomolecules. (2024) 14:1646. doi: 10.3390/biom14121646, PMID: 39766353 PMC11674224

[78] Ai K Li K Jiao X Zhang Y Li J Zhang Q. IL-2-mTORC1 signaling coordinates the STAT1/T-bet axis to ensure Th1 cell differentiation and anti-bacterial immune response in fish. PLoS Pathog. (2022) 18:e1010913. doi: 10.1371/journal.ppat.1010913, PMID: 36282845 PMC9595569

[79] Martín-Sierra C Martins R Coucelo M Abrantes AM Caetano Oliveira R Tralhão JG. Tumor resection in hepatic carcinomas restores circulating T regulatory cells. J Clin Med. (2024) 13:6011. doi: 10.3390/jcm13196011, PMID: 39408071 PMC11478317

[80] Zhang X Liang C Wu C Wan S Xu L Wang S. A rising star involved in tumour immunity: Lactylation. J Cell Mol Med. (2024) 28:e70146. doi: 10.1111/jcmm.70146, PMID: 39417674 PMC11483924

[81] Lopez Krol A Nehring HP Krause FF Wempe A Raifer H Nist A. Lactate induces metabolic and epigenetic reprogramming of pro-inflammatory Th17 cells. EMBO Rep. (2022) 23:e54685. doi: 10.15252/embr.202254685, PMID: 36215678 PMC9724659

[82] Tong H Jiang Z Song L Tan K Yin X He C. Dual impacts of serine/glycine-free diet in enhancing antitumor immunity and promoting evasion via PD-L1 lactylation. Cell Metab. (2024) 36:2493–2510.e9. doi: 10.1016/j.cmet.2024.10.019, PMID: 39577415

[83] Gu J Zhou J Chen Q Xu X Gao J Li X. Tumor metabolite lactate promotes tumorigenesis by modulating MOESIN lactylation and enhancing TGF-β signaling in regulatory T cells. Cell Rep. (2022) 39:110986. doi: 10.1016/j.celrep.2022.110986, PMID: 35732125

[84] Liu Y Wang F Peng D Zhang D Liu L Wei J. Activation and antitumor immunity of CD8(+) T cells are supported by the glucose transporter GLUT10 and disrupted by lactic acid. Sci Transl Med. (2024) 16:eadk7399. doi: 10.1126/scitranslmed.adk7399, PMID: 39196962

[85] Nicolini A Ferrari P. Involvement of tumor immune microenvironment metabolic reprogramming in colorectal cancer progression, immune escape, and response to immunotherapy. Front Immunol. (2024) 15:1353787. doi: 10.3389/fimmu.2024.1353787, PMID: 39119332 PMC11306065

[86] Deng H Kan A Lyu N He M Huang X Qiao S. Tumor-derived lactate inhibit the efficacy of lenvatinib through regulating PD-L1 expression on neutrophil in hepatocellular carcinoma. J Immunother Cancer. (2021) 9:e002305. doi: 10.1136/jitc-2020-002305, PMID: 34168004 PMC8231064

[87] Yi M Niu M Xu L Luo S Wu K. Regulation of PD-L1 expression in the tumor microenvironment. J Hematol OncolJ Hematol Oncol. (2021) 14:10. doi: 10.1186/s13045-020-01027-5, PMID: 33413496 PMC7792099

[88] Mittendorf EA Philips AV Meric-Bernstam F Qiao N Wu Y Harrington S. PD-L1 expression in triple-negative breast cancer. Cancer Immunol Res. (2014) 2:361–70. doi: 10.1158/2326-6066.CIR-13-0127, PMID: 24764583 PMC4000553

[89] Wang Y Zhang C Yan M Ma X Song L Wang B. PD-L1 regulates tumor proliferation and T-cell function in NF2-associated meningiomas. CNS Neurosci Ther. (2024) 30:e14784. doi: 10.1111/cns.14784, PMID: 38828669 PMC11145367

[90] Atefi M Avramis E Lassen A Wong DJL Robert L Foulad D. Effects of MAPK and PI3K pathways on PD-L1 expression in melanoma. Clin Cancer Res Off J Am Assoc Cancer Res. (2014) 20:3446–57. doi: 10.1158/1078-0432.CCR-13-2797, PMID: 24812408 PMC4079734

[91] Tang X Yang J Shi A Xiong Y Wen M Luo Z. CD155 cooperates with PD-1/PD-L1 to promote proliferation of esophageal squamous cancer cells via PI3K/akt and MAPK signaling pathways. Cancers. (2022) 14:5610. doi: 10.3390/cancers14225610, PMID: 36428703 PMC9688614

[92] Huang Z-W Zhang X-N Zhang L Liu L-L Zhang J-W Sun Y-X. STAT5 promotes PD-L1 expression by facilitating histone lactylation to drive immunosuppression in acute myeloid leukemia. Signal Transduct Target Ther. (2023) 8:391. doi: 10.1038/s41392-023-01605-2, PMID: 37777506 PMC10542808

[93] Kythreotou A Siddique A Mauri FA Bower M Pinato DJ. PD-L1. J Clin Pathol. (2018) 71:189–94. doi: 10.1136/jclinpath-2017-204853, PMID: 29097600

[94] Raychaudhuri D Singh P Chakraborty B Hennessey M Tannir AJ Byregowda S. Histone lactylation drives CD8(+) T cell metabolism and function. Nat Immunol. (2024) 25:2140–51. doi: 10.1038/s41590-024-01985-9, PMID: 39375549 PMC13211864

[95] Wang R Li C Cheng Z Li M Shi J Zhang Z. H3K9 lactylation in Malignant cells facilitates CD8(+) T cell dysfunction and poor immunotherapy response. Cell Rep. (2024) 43:114686. doi: 10.1016/j.celrep.2024.114686, PMID: 39216002

[96] Sun K Zhang X Shi J Huang J Wang S Li X. Elevated protein lactylation promotes immunosuppressive microenvironment and therapeutic resistance in pancreatic ductal adenocarcinoma. J Clin Invest. (2025) 135:e187024. doi: 10.1172/JCI187024, PMID: 39883522 PMC11957693

[97] Huang H Chen K Zhu Y Hu Z Wang Y Chen J. A multi-dimensional approach to unravel the intricacies of lactylation related signature for prognostic and therapeutic insight in colorectal cancer. J Transl Med. (2024) 22:211. doi: 10.1186/s12967-024-04955-9, PMID: 38419085 PMC10900655

[98] Ma Z Yang J Jia W Li L Li Y Hu J. Histone lactylation-driven B7-H3 expression promotes tumor immune evasion. Theranostics. (2025) 15:2338–59. doi: 10.7150/thno.105947, PMID: 39990209 PMC11840737

[99] Watson MJ Vignali PDA Mullett SJ Overacre-Delgoffe AE Peralta RM Grebinoski S. Metabolic support of tumour-infiltrating regulatory T cells by lactic acid. Nature. (2021) 591:645–51. doi: 10.1038/s41586-020-03045-2, PMID: 33589820 PMC7990682

[100] MaruYama T Kobayashi S Shibata H Chen W Owada Y. Curcumin analog GO-Y030 boosts the efficacy of anti-PD-1 cancer immunotherapy. Cancer Sci. (2021) 112:4844–52. doi: 10.1111/cas.15136, PMID: 34529884 PMC8645716

[101] Chen J Zhao D Wang Y Liu M Zhang Y Feng T. Lactylated apolipoprotein C-II induces immunotherapy resistance by promoting extracellular lipolysis. Adv Sci Weinh Baden-Wurtt Ger. (2024) 11:e2406333. doi: 10.1002/advs.202406333, PMID: 38981044 PMC11481198

[102] Su J Mao X Wang L Chen Z Wang W Zhao C. Lactate/GPR81 recruits regulatory T cells by modulating CX3CL1 to promote immune resistance in a highly glycolytic gastric cancer. Oncoimmunology. (2024) 13:2320951. doi: 10.1080/2162402X.2024.2320951, PMID: 38419759 PMC10900271

[103] Metelitsa LS. Anti-tumor potential of type-I NKT cells against CD1d-positive and CD1d-negative tumors in humans. Clin Immunol Orlando Fla. (2011) 140:119–29. doi: 10.1016/j.clim.2010.10.005, PMID: 21095162 PMC3444285

[104] Bayatipoor H Mehdizadeh S Jafarpour R Shojaei Z Pashangzadeh S Motallebnezhad M. Role of NKT cells in cancer immunotherapy-from bench to bed. Med Oncol Northwood Lond Engl. (2022) 40:29. doi: 10.1007/s12032-022-01888-5, PMID: 36460881

[105] Terabe M Berzofsky JA. The immunoregulatory role of type I and type II NKT cells in cancer and other diseases. Cancer Immunol Immunother CII. (2014) 63:199–213. doi: 10.1007/s00262-013-1509-4, PMID: 24384834 PMC4012252

[106] Petrovic A Jovanovic I Stojanovic B Dimitrijevic Stojanovic M Stojanovic BS Jurisevic M. Harnessing metformin’s immunomodulatory effects on immune cells to combat breast cancer. Int J Mol Sci. (2024) 25:5869. doi: 10.3390/ijms25115869, PMID: 38892058 PMC11172298

[107] Lam PY Kobayashi T Soon M Zeng B Dolcetti R Leggatt G. NKT cell-driven enhancement of antitumor immunity induced by clec9a-targeted tailorable nanoemulsion. Cancer Immunol Res. (2019) 7:952–62. doi: 10.1158/2326-6066.CIR-18-0650, PMID: 31053598

[108] Wang Z-H Zhang P Peng W-B Ye L-L Xiang X Wei X-S. Altered phenotypic and metabolic characteristics of FOXP3+ CD3+ CD56+ natural killer T (NKT)-like cells in human Malignant pleural effusion. OncoImmunology. (2023) 12:2160558. doi: 10.1080/2162402X.2022.2160558, PMID: 36567801 PMC9788685

[109] Xu Y Hao X Ren Y Xu Q Liu X Song S. Research progress of abnormal lactate metabolism and lactate modification in immunotherapy of hepatocellular carcinoma. Front Oncol. (2023) 12:1063423. doi: 10.3389/fonc.2022.1063423, PMID: 36686771 PMC9853001

[110] Reid FSW Egoroff N Pockney PG Smith SR. A systematic scoping review on natural killer cell function in colorectal cancer. Cancer Immunol Immunother CII. (2021) 70:597–606. doi: 10.1007/s00262-020-02721-6, PMID: 32918127 PMC10992123

[111] Enomoto Y Li P Jenkins LM Anastasakis D Lyons GC Hafner M. Cytokine-enhanced cytolytic activity of exosomes from NK Cells. Cancer Gene Ther. (2022) 29:734–49. doi: 10.1038/s41417-021-00352-2, PMID: 34316033 PMC9209332

[112] Kumar B Singh A Basar R Uprety N Li Y Fan H. BATF is a major driver of NK cell epigenetic reprogramming and dysfunction in AML. Sci Transl Med. (2024) 16:eadp0004. doi: 10.1126/scitranslmed.adp0004, PMID: 39259809 PMC11967735

[113] Roe K. NK-cell exhaustion, B-cell exhaustion and T-cell exhaustion-the differences and similarities. Immunology. (2022) 166:155–68. doi: 10.1111/imm.13464, PMID: 35266556

[114] Vivier E Tomasello E Baratin M Walzer T Ugolini S. Functions of natural killer cells. Nat Immunol. (2008) 9:503–10. doi: 10.1038/ni1582, PMID: 18425107

[115] Zhang Y Zhai Z Duan J Wang X Zhong J Wu L. Lactate: the mediator of metabolism and immunosuppression. Front Endocrinol. (2022) 13:901495. doi: 10.3389/fendo.2022.901495, PMID: 35757394 PMC9218951

[116] Tang F Xiao D Li X Qiao L. The roles of lactate and the interplay with m6A modification in diseases. Cell Biol Toxicol. (2024) 40:107. doi: 10.1007/s10565-024-09951-9, PMID: 39617813 PMC11609124

[117] Wu Q Li X Long M Xie X Liu Q. Integrated analysis of histone lysine lactylation (Kla)-specific genes suggests that NR6A1, OSBP2 and UNC119B are novel therapeutic targets for hepatocellular carcinoma. Sci Rep. (2023) 13:18642. doi: 10.1038/s41598-023-46057-4, PMID: 37903971 PMC10616101

[118] Herrera FG Ronet C Ochoa de Olza M Barras D Crespo I Andreatta M. Low-dose radiotherapy reverses tumor immune desertification and resistance to immunotherapy. Cancer Discov. (2022) 12:108–33. doi: 10.1158/2159-8290.CD-21-0003, PMID: 34479871 PMC9401506

[119] Wu Z Wu H Dai Y Wang Z Han H Shen Y. A pan-cancer multi-omics analysis of lactylation genes associated with tumor microenvironment and cancer development. Heliyon. (2024) 10:e27465. doi: 10.1016/j.heliyon.2024.e27465, PMID: 38463768 PMC10923869

[120] Garcia-Carpizo V Ruiz-Llorente S Sarmentero J González-Corpas A Barrero MJ. CREBBP/EP300 bromodomain inhibition affects the proliferation of AR-positive breast cancer cell lines. Mol Cancer Res MCR. (2019) 17:720–30. doi: 10.1158/1541-7786.MCR-18-0719, PMID: 30606771

[121] Hu T Cheng B Matsunaga A Zhang T Lu X Fang H. Single-cell analysis defines highly specific leukemia-induced neutrophils and links MMP8 expression to recruitment of tumor associated neutrophils during FGFR1 driven leukemogenesis. Exp Hematol Oncol. (2024) 13:49. doi: 10.1186/s40164-024-00514-6, PMID: 38730491 PMC11084112

[122] Zhang J Gu J Wang X Ji C Yu D Wang M. Engineering and targeting neutrophils for cancer therapy. Adv Mater Deerfield Beach Fla. (2024) 36:e2310318. doi: 10.1002/adma.202310318, PMID: 38320755

[123] Ugolini A De Leo A Yu X Scirocchi F Liu X Peixoto B. Functional reprogramming of neutrophils within the brain tumor microenvironment by hypoxia-driven histone lactylation. Cancer Discov. (2025) 15:1270–96. doi: 10.1158/2159-8290.CD-24-1056, PMID: 40014923 PMC12133432

[124] Kumar V Patel S Tcyganov E Gabrilovich DI. The nature of myeloid-derived suppressor cells in the tumor microenvironment. Trends Immunol. (2016) 37:208–20. doi: 10.1016/j.it.2016.01.004, PMID: 26858199 PMC4775398

[125] Kaczmarek F Marcinkowska-Gapińska A Bartkowiak-Wieczorek J Nowak M Kmiecik M Brzezińska K. Blood-based biomarkers as predictive and prognostic factors in immunotherapy-treated patients with solid tumors-currents and perspectives. Cancers. (2025) 17:2001. doi: 10.3390/cancers17122001, PMID: 40563651 PMC12190272

[126] Kumar V Cheng P Condamine T Mony S Languino LR McCaffrey JC. CD45 phosphatase inhibits STAT3 transcription factor activity in myeloid cells and promotes tumor-associated macrophage differentiation. Immunity. (2016) 44:303–15. doi: 10.1016/j.immuni.2016.01.014, PMID: 26885857 PMC4759655

[127] Noman MZ Janji B Hu S Wu JC Martelli F Bronte V. Tumor-promoting effects of myeloid-derived suppressor cells are potentiated by hypoxia-induced expression of miR-210. Cancer Res. (2015) 75:3771–87. doi: 10.1158/0008-5472.CAN-15-0405, PMID: 26206559

[128] Yang X Lu Y Hang J Zhang J Zhang T Huo Y. Lactate-modulated immunosuppression of myeloid-derived suppressor cells contributes to the radioresistance of pancreatic cancer. Cancer Immunol Res. (2020) 8:1440–51. doi: 10.1158/2326-6066.CIR-20-0111, PMID: 32917658

[129] Lundø K Trauelsen M Pedersen SF Schwartz TW. Why warburg works: lactate controls immune evasion through GPR81. Cell Metab. (2020) 31:666–8. doi: 10.1016/j.cmet.2020.03.001, PMID: 32268113

[130] Li F Si W Xia L Yin D Wei T Tao M. Positive feedback regulation between glycolysis and histone lactylation drives oncogenesis in pancreatic ductal adenocarcinoma. Mol Cancer. (2024) 23:90. doi: 10.1186/s12943-024-02008-9, PMID: 38711083 PMC11071201

[131] Deng J Liao X. Lysine lactylation (Kla) might be a novel therapeutic target for breast cancer. BMC Med Genomics. (2023) 16:283. doi: 10.1186/s12920-023-01726-1, PMID: 37950222 PMC10636881

[132] Umar SM Dev AJR Kashyap A Rathee M Chauhan SS Sharma A. 7-amino carboxycoumarin 2 inhibits lactate induced epithelial-to-mesenchymal transition via MPC1 in oral and breast cancer cells. Cell Biol Int. (2024) 48:1185–97. doi: 10.1002/cbin.12172, PMID: 38773713

[133] Li W Zhou C Yu L Hou Z Liu H Kong L. Tumor-derived lactate promotes resistance to bevacizumab treatment by facilitating autophagy enhancer protein RUBCNL expression through histone H3 lysine 18 lactylation (H3K18la) in colorectal cancer. Autophagy. (2024) 20:114–30. doi: 10.1080/15548627.2023.2249762, PMID: 37615625 PMC10761097

[134] Sonveaux P Copetti T De Saedeleer CJ Végran F Verrax J Kennedy KM. Targeting the lactate transporter MCT1 in endothelial cells inhibits lactate-induced HIF-1 activation and tumor angiogenesis. PLoS One. (2012) 7:e33418. doi: 10.1371/journal.pone.0033418, PMID: 22428047 PMC3302812

[135] Verma S Budhu S Serganova I Dong L Mangarin LM Khan JF. Pharmacologic LDH inhibition redirects intratumoral glucose uptake and improves antitumor immunity in solid tumor models. J Clin Invest. (2024) 134:e177606. doi: 10.1172/JCI177606, PMID: 39225102 PMC11364391

[136] Babl N Decking S-M Voll F Althammer M Sala-Hojman A Ferretti R. MCT4 blockade increases the efficacy of immune checkpoint blockade. J Immunother Cancer. (2023) 11:e007349. doi: 10.1136/jitc-2023-007349, PMID: 37880183 PMC10603342

[137] Fang Y Liu W Tang Z Ji X Zhou Y Song S. Monocarboxylate transporter 4 inhibition potentiates hepatocellular carcinoma immunotherapy through enhancing T cell infiltration and immune attack. Hepatol Baltim Md. (2023) 77:109–23. doi: 10.1002/hep.32348, PMID: 35043976

[138] Zhang Y-T Xing M-L Fang H-H Li W-D Wu L Chen Z-P. Effects of lactate on metabolism and differentiation of CD4(+)T cells. Mol Immunol. (2023) 154:96–107. doi: 10.1016/j.molimm.2022.12.015, PMID: 36621062

[139] Zong Z. Expanding the genetic code for site-specific lysine lactylation. Nat Rev Mol Cell Biol. (2025) 26:252–2. doi: 10.1038/s41580-025-00832-5, PMID: 39905193

